# Cardiac arrhythmias and conduction abnormalities in patients with type 2 diabetes

**DOI:** 10.1038/s41598-023-27941-5

**Published:** 2023-01-21

**Authors:** Araz Rawshani, Darren K. McGuire, Elmir Omerovic, Naveed Sattar, John J. V. McMurray, Ulf Smith, Bjorn Redfors, Lennart Bergfeldt, Bjorn Eliasson, Jan Borén, Deepak L. Bhatt, Goran Bergstrom, Aidin Rawshani

**Affiliations:** 1 Department of Molecular and Clinical Medicine, Institute of Medicine, University of Gothenburg, Göteborg, Sweden; 2 Wallenberg Laboratory for Cardiovascular and Metabolic Research, Institute of Medicine, University of Gothenburg, Göteborg, Sweden; 3 Department of Internal Medicine, University of Texas Southwestern Medical Center, and Parkland Health and Hospital System, Dallas, USA; 4 Institute of Cardiovascular and Medical Sciences, British Heart Foundation Glasgow Cardiovascular Research Centre, Glasgow, UK; 5 The Lundberg Laboratory for Diabetes Research, Department of Molecular and Clinical Medicine, Sahlgrenska University Hospital, Göteborg, Sweden; 6 Brigham and Women’s Hospital Heart and Vascular Center, Harvard Medical School, Boston, MA USA

**Keywords:** Biomarkers, Cardiology, Endocrinology, Medical research, Risk factors

## Abstract

The association between type 2 diabetes (T2D) and the development of cardiac arrhythmias and conduction disturbances has not been extensively studied. Arrhythmia was defined as atrial fibrillation and flutter (AF/AFl), ventricular tachycardia (VT) and ventricular fibrillation (VF), and conduction abnormality as sinus node disease (SND), atrioventricular (AV) block or pacemaker implantation, and intraventricular conduction blocks (IVCB). Incidence rates and Cox regression were used to compare outcomes, and to assess optimal levels for cardiometabolic risk factors and risk associated with multifactorial risk factor control (i.e., HbA1c, LDL-C, systolic blood pressure (SBP), BMI and eGFR), between patients with versus without T2D. The analyses included data from 617,000 patients with T2D and 2,303,391 matched controls. Patients with diabetes and the general population demonstrated a gradual increase in rates for cardiac conduction abnormalities and virtually all age-groups for AF/AFI showed increased incidence during follow-up. For patients with versus without T2D, risks for cardiac arrhythmias were higher, including for AF/AFl (HR 1.17, 95% CI 1.16–1.18), the composite of SND, AV-block or pacemaker implantation (HR 1.40, 95% CI 1.37–1.43), IVCB (HR 1.23, 95% CI 1.18–1.28) and VT/VF (HR 1.08, 95% CI 1.04–1.13). For patients with T2D who had selected cardiometabolic risk factors within target ranges, compared with controls, risk of arrythmia and conduction abnormalities for T2D vs not were: AF/AFl (HR 1.09, 95% CI 1.05–1.14), the composite of SND, AV-block or pacemaker implantation (HR 1.06, 95% CI 0.94–1.18), IVCB (HR 0.80, 95% CI 0.60–0.98), and for VT/VF (HR 0.97, 95% CI 0.80–1.17). Cox models showed a linear risk increase for SBP and BMI, while eGFR showed a U-shaped association. Individuals with T2D had a higher risk of arrhythmias and conduction abnormalities than controls, but excess risk associated with T2D was virtually not evident among patients with T2D with all risk factors within target range. BMI, SBP and eGFR displayed significant associations with outcomes among patients with T2D.

## Introduction

The association between type 2 diabetes (T2D) and the premature development of coronary artery disease and myocardial disease is well described, yet much less is known about the possible impact of T2D on cardiac conduction and risk for arrhythmias. Clearly, myocardial ischaemia, myocardial infarction and ischemic cardiomyopathy contribute to increased risk for ventricular arrhythmias, but other possible contributors to increased arrhythmia risk in the setting of T2D include chronic hyperglycaemia, systemic inflammation, and oxidative stress that individually and additively may contribute to myocardial fibrosis, including in the atria, ventricles and conducting tissues. Along with autonomic neuropathy and electrolyte abnormalities commonly associated with T2D, this complex milieu may lead to a variety of cardiac electrical complications.

While the risks of chronic and acute coronary syndromes, heart failure, kidney failure and stroke have been reduced dramatically over recent decades^1^, few studies have addressed trends in arrhythmias in people with and without T2D^2,3^.

In this nationwide observational study, long-term trends of atrial fibrillation and flutter (AF/AFl), sinus node disease (SND), atrioventricular (AV) block, intraventricular conduction block (IVCB), ventricular tachycardia (VT) and ventricular fibrillation (VF), were examined in people with T2D from the Swedish National Diabetes Registry, compared with controls from the general population. Moreover, the association between multifactorial risk factor control and optimal levels were examined for selected cardiovascular risk factors and each outcome.

## Methods

Data are available from the sources stated in the paper on request to the data providers, fulfilling legal and regulatory requirements and with permission from the Swedish Ethical Review Authority of Ministry of Sweden. The first and last author had full access to all the data in the study and take responsibility for its integrity and the data analyses. All methods were carried out in accordance with relevant guidelines and regulations.

### Study design and support

The study was approved by the Ethical Review Authority of Ministry of Sweden. All patients provided informed consent before inclusion in the registry. The Swedish Heart and Lung Foundation, the Swedish state under an agreement between the Swedish government and the county councils concerning economic support of research and education of doctors and The Swedish Research Council funded the study; no industry support was provided.

### Data sources and study cohort

The Swedish National Diabetes Registry (NDR) has been described previously^1,4,5^. The distinction for T2D is based on the epidemiological definition and the clinical evaluation. Patients with at least one entry in the registry between Jan 1st 2001 to Dec 31st 2019, were included in the study. At enrollment in the registry, each person with T2D older than 18 years of age in Sweden, was matched for age, sex and county of residence, with roughly five controls without diabetes. The controls were randomly selected from the Swedish population register by Statistics Sweden. Patients with T2D that fulfilled any exclusion criterion, were excluded along with all their matched controls, whereas, controls that fulfilled an exclusion criterion, were excluded separately.

### Outcomes

**Figure 1:**
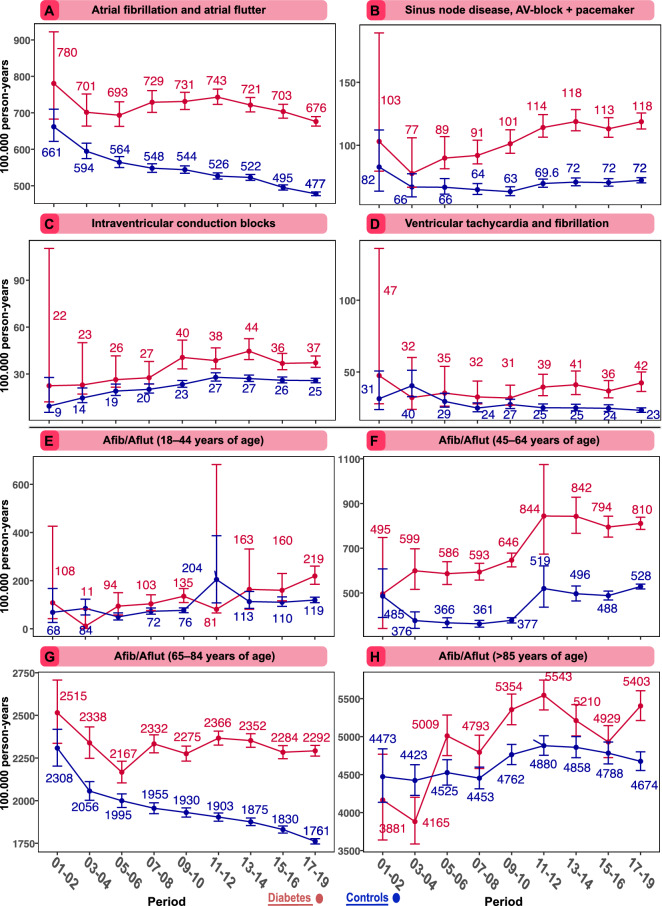
Standardized incidence rates for cardiac arrhythmias among patients with type 2 diabetes, as well as, matched controls from general population, per 10. Panel E–H display atrial fibrillation and flutter among various age-categories. The dark lines indicate the hazard function and the shaded areas 95% confidence intervals.

Excess risk and long-term trends were assessed (patients with diabetes vs. controls) with regards to four composite outcomes, (1) atrial fibrillation and/or atrial flutter (AF, AFl); (2) sinus node disease (SND), atrioventricular block (AVB) or pacemaker implantation; (3) intraventricular conduction blocks (IVCB), i.e., right bundle branch block, left bundle branch block, left anterior fascicular block, left posterior fascicular block, bifascicular block or trifascicular block; and (4) ventricular tachycardia and/or fibrillation (VT, VF). Outcomes were retrieved from inpatient and outpatient records with the use of codes from the International Classification of Disease (ICD) version 9 and 10. The main diagnosis and up to six secondary diagnosis codes were used to identify outcomes in the Swedish inpatient- and outpatient registry. The specific codes are listed in Supplementary Table S1. Patients were followed until an event, death, or December 31, 2019. For the incidence analyses (Fig. 1), we performed a stratified analyses between different age-groups to further investigate long-term trends for AF/AFI.

### Statistical analyses

#### Excess risk in patients with diabetes and long-term trends

Incidence rates were calculated with direct standardization and reported as the number of events per 100,000 person-years. The study period (2001–2019) was divided into 2-year intervals, with the exception of the final period, which was a 3-year period. The incidence for each interval was standardized to the age- and sex distribution of the first time period. Numerators were the number of incident events each time period, while denominators were the number of persons at risk during the same time period. The number of events and person-years for each time period and outcome are presented in Tables S2 and S3. These tables also include information on crude- and standardized incidence rates. In Table 1, results from Cox regression are presented for changes in risk over time for all outcomes, among patients with diabetes and controls. The regression models included age, sex, time-updated time periods, and for some models, interactions terms.

**Table 1:** Baseline characteristics for patients with type 2 diabetes, along with matched controls from the general population.

	Type 2 diabetes	Matched controls	Diabetes < 45	Diabetes 45–54	Diabetes 55–64	Diabetes 65–74	Diabetes > 75
Number of study participants	617,000	2,303,391	43,599	87,665	196,498	168,450	120,788
Sex = Women (%)	271,092 (43.9)	1,108,812 (48.1)	18,447 (42.3)	33,049 (37.7)	79,092 (40.3)	74,420 (44.2)	66,084 (54.7)
Age (mean (SD))	63.61 (12.45)	61.29 (12.53)	37.58 (6.15)	50.12 (2.81)	60.15 (2.62)	69.25 (2.84)	80.59 (4.57)
< 45	43,599 (7.1)	219,045 (9.5)					
45–54	87,665 (14.2)	403,697 (17.5)					
55–64	196,498 (31.8)	790,319 (34.3)					
65–74	168,450 (27.3)	553,558 (24.0)					
> 75	120,788 (19.6)	336,772 (14.6)					
Education (%)							
Pre-secondary education ≤ 9 years	268,392 (43.5)	785,639 (34.1)	12,190 (28.0)	22,502 (25.7)	87,786 (44.7)	73,716 (43.8)	72,198 (59.8)
Secondary education > 9 to 12 years	244,652 (39.7)	910,017 (39.5)	21,883 (50.2)	45,827 (52.3)	75,058 (38.2)	66,254 (39.3)	35,630 (29.5)
Post-secondary education ≥ 12 years	103,956 (16.8)	607,735 (26.4)	9526 (21.8)	19,336 (22.1)	33,654 (17.1)	28,480 (16.9)	12,960 (10.7)
Marital status (%)							
Married	308,334 (50.0)	1,154,156 (50.1)	17,786 (40.8)	43,574 (49.7)	90,005 (45.8)	100,217 (59.5)	56,752 (47.0)
Ethnicity = Scandinavia (%)	512,336 (83.0)	2,052,023 (89.1)	28,078 (64.4)	63,552 (72.5)	157,999 (80.4)	150,981 (89.6)	111,726 (92.5)
Income family (IQR) (%)							
IQR 1	182,936 (29.6)	520,554 (22.6)	12,165 (27.9)	19,660 (22.4)	35,806 (18.2)	52,432 (31.1)	62,873 (52.1)
IQR 2	173,826 (28.2)	550,836 (23.9)	12,508 (28.7)	22,244 (25.4)	42,619 (21.7)	55,706 (33.1)	40,749 (33.7)
IQR 3	149,168 (24.2)	589,482 (25.6)	9787 (22.4)	19,853 (22.6)	74,894 (38.1)	33,582 (19.9)	11,052 (9.1)
IQR4	111,070 (18.0)	642,519 (27.9)	9139 (21.0)	25,908 (29.6)	43,179 (22.0)	26,730 (15.9)	6114 (5.1)
Income (IQR) (%)							
IQR 1	174,838 (28.3)	494,745 (21.5)	12,552 (28.8)	18,509 (21.1)	34,478 (17.5)	53,408 (31.7)	55,891 (46.3)
IQR 2	178,929 (29.0)	532,630 (23.1)	10,846 (24.9)	21,283 (24.3)	41,167 (21.0)	58,913 (35.0)	46,720 (38.7)
IQR 3	146,088 (23.7)	606,775 (26.3)	10,589 (24.3)	22,210 (25.3)	75,183 (38.3)	27,108 (16.1)	10,998 (9.1)
IQR4	117,145 (19.0)	669,241 (29.1)	9612 (22.0)	25,663 (29.3)	45,670 (23.2)	29,021 (17.2)	7179 (5.9)
Coronary heart disease = Yes (%)	81,785 (13.3)	121,506 (5.3)	624 (1.4)	5273 (6.0)	22,087 (11.2)	27,695 (16.4)	26,106 (21.6)
Prior myocardial infarction = Yes (%)	38,462 (6.2)	52,348 (2.3)	353 (0.8)	2906 (3.3)	10,800 (5.5)	12,521 (7.4)	11,882 (9.8)
Stroke = Yes (%)	29,462 (4.8)	56,678 (2.5)	267 (0.6)	1518 (1.7)	6773 (3.4)	9651 (5.7)	11,253 (9.3)
Heart failure = Yes (%)	22,596 (3.7)	33,217 (1.4)	301 (0.7)	1375 (1.6)	4845 (2.5)	6576 (3.9)	9499 (7.9)
Hypertension = Yes (%)	162,063 (26.3)	248,628 (10.8)	3520 (8.1)	14,662 (16.7)	48,447 (24.7)	52,293 (31.0)	43,141 (35.7)
Peripheral arterial disease = Yes (%)	11,501 (1.9)	17,406 (0.8)	39 (0.1)	372 (0.4)	2411 (1.2)	4031 (2.4)	4648 (3.8)
Chronic obstructive pulmonary disease = Yes (%)	15,758 (2.6)	39,749 (1.7)	104 (0.2)	837 (1.0)	4248 (2.2)	5889 (3.5)	4680 (3.9)
Dementia = Yes (%)	4249 (0.7)	23,650 (1.0)	12 (0.0)	49 (0.1)	431 (0.2)	943 (0.6)	2814 (2.3)
Alcoholism = Yes (%)	16,715 (2.7)	53,702 (2.3)	1565 (3.6)	3675 (4.2)	6748 (3.4)	3819 (2.3)	908 (0.8)
End-stage kidney disease = Yes (%)	12,492 (2.1)	17,021 (0.9)	551 (1.4)	1235 (1.5)	3313 (1.8)	3642 (2.2)	3751 (3.1)
Antihypertensive medication = Yes (%)	364,740 (59.1)	791,438 (34.4)	19,513 (44.8)	57,631 (65.7)	136,157 (69.3)	107,823 (64.0)	43,616 (36.1)
Statin = Yes (%)	310,954 (50.4)	378,603 (16.4)	20,162 (46.2)	54,123 (61.7)	119,813 (61.0)	89,260 (53.0)	27,596 (22.8)
Anti-coagulant medication = Yes (%)	54,791 (8.9)	149,191 (6.5)	1279 (2.9)	4855 (5.5)	17,283 (8.8)	20,829 (12.4)	10,545 (8.7)
Antithrombotic medication = Yes (%)	149,563 (24.2)	270,257 (11.7)	3945 (9.0)	18,103 (20.7)	54,126 (27.5)	50,215 (29.8)	23,174 (19.2)
Imputed baseline values for individuals with diabetes							
Insulin method = 2 (%)	2608 (0.4)						
Age of onset of disease (y, mean (SD))	58.8 (14.8)						
Duration of diabetes (y, mean (SD))	3.8 (6.1)						
Glycated haemoglobin (HbA1c) (mmol/L; mean (SD))*	55.18 (17.01)						
Smoker = Yes (%)	104,717 (17.0)						
Albuminuria (%)							
No albuminuria	494,607 (80.2)						
Normalized value	2690 (0.4)						
Microalbuminuria (3–30)	83,597 (13.5)						
Macroalbuminuria (> 30)	36,106 (5.9)						
eGFR (mL/min/1.73 m^2^, mean (SD))	85.2 (28.0)						
Retinopathy = Yes (%)	107,648 (17.4)						
Systolic blood pressure (mmHg, mean (SD))	138.2 (17.4)						
Diastolic blood pressure (mmHg, mean (SD))	79.4 (9.99)						
Total cholesterol (mmol/L, mean (SD))	5.09 (1.16)						
High-density lipoprotein cholesterol (mmol/L, mean (SD))	1.28 (0.43)						
Triglycerides (mmol/L, mean (SD))	2.04 (1.52)						
LDL-cholesterol (mmol/L, mean (SD))	2.95 (0.99)						
S-creatinine (mg/dL, mean (SD))	77.4 (27.4)						
Body mass index (kg/m^2^, mean (SD))	30.27 (5.63)						
Physical activity (%)							
Never	89,573 (14.5)						
< 1 time/week	91,688 (14.9)						
1–2 time/week	123,238 (20.0)						
3–5 time/week	133,085 (21.6)						
5 time/week	179,416 (29.1)						

Values are mean (SD).

Income and eGFR is reported as median (inter quartile range [IQR]).

Glomerular filtration rate was estimated using the Modification of Diet in Renal Disease Study Equation.

Controls are individuals, matched for age, sex and county, who were randomly selected from the general population.

*Concentrations of glycated haemoglobin are based on values from the International Federation of Clinical Chemistry.

#### Association between risk factors and arrhythmia outcomes

Cox regression analyses were used to analyze the associations between cardiometabolic risk markers and risk of each arrhythmia in patients with T2D. Metabolic markers were not available for the controls, and were therefore not included in the modeling. Age, glycated haemoglobin (mmol/mol), body mass index (BMI, kg/m^2^), systolic blood pressure (mmHg), diastolic blood pressure (mmHg), low-density lipoprotein (LDL-C; mmol/L) cholesterol (mmol/L), high-density lipoprotein (HDL-C; mmol/L) cholesterol, estimated glomerular filtration rate (eGFR mL/min/1.72 m^2^) and duration of diabetes (years), were modelled using restricted cubic splines with 3 knots to capture non-linear associations. Covariate adjustment was made for age, sex, smoking, physical activity, ethnicity, marital status, income and educational level, comorbidities and pharmacological treatment of diabetes. These models also allowed for the identification of the risk factor level associated with the lowest risk of each arrhythmia outcome. In addition, regression models were constructed to assess the relationship of age at baseline and for those with T2D, the duration of diabetes.

#### Multifactorial risk factor control

The associations between multifactorial CV risk factor control and risk of arrhythmia were examined. This was done among patients with diabetes by stratifying them according to the number of CV risk factors not within target ranges (guideline-recommended target levels). The following five risk factors were considered (cut-offs in parentheses): glycated haemoglobin (≥ 7.0% [≥ 53 mmol/mol]), systolic and diastolic blood pressure (either ≥ 130 mmHg systolic or ≥ 80 diastolic), micro- or macroalbuminuria), smoking (being a current smoker at study entry) and LDL-C (≥ 2.5 mmol/L [97 mg per deciliter]). Consistent with prior analyses of this dataset^5^, adjustment for duration of diabetes was done by assigning matched controls to a duration of zero years and patients with diabetes had their duration of diabetes centralized around the grand mean.

Missing data (around 5–10%) were handled using multiple imputations by chained equations (MICE). Distributions and means were analyzed before and after imputation without observing any material difference. All variables that were used in the imputation model are presented in Table S4. Instead of using p-values and adjusting for multiple hypothesis tests, statistical significance was based upon confidence intervals overlapping with 1.0. A comprehensive discussion of statistical methods and model construction has been presented in the supplementary material. Calculations were performed in R version 4.0.3 (R Foundation for Statistical Computing), using RStudio.

## Results

### Study population

The study cohort comprised 617,000 patients with T2D and 2,303,391 matched controls. The mean age was 63 years for participants with type 2 diabetes. Coexisting conditions were approximately twice as frequent in people with diabetes. Median follow-up for patients with type 2 diabetes was 7.6 years (Table 1).

### Incidence rates and long-term trends.

#### Atrial fibrillation and/or flutter

In people with type 2 diabetes, the incidence rates of AF/AFl decreased slightly from 780 to 676 cases per 100,000 person-years from the first to final time period. Corresponding figures for controls were 661 to 477 cases per 100,000 person-years (Fig. 1A). Patients with type 2 diabetes displayed roughly a 30% higher risk for atrial fibrillation and flutter, in the first- and last time periods, compared with matched controls. There was no significant change in incremental risk over time between patients with diabetes and controls (Table 2). Hazard ratio for a 10-year period showed a 5% risk reduction for atrial fibrillation and flutter (HR 0.95, 95% CI 0.93–0.98) for patients with diabetes and 9% risk reduction for controls (Table 2).

**Table 2:** Cox regression to estimate change in risk for cardiac arrhythmias in patients with type 2 diabetes and matched controls.

Outcomes	^a^Diabetes vs controls (Period 1–3)	^a^Diabetes vs controls (Period 7–9)	^b^Diabetes vs controls (Period 1–3 vs 7–9)
Atrial fibrillation and flutter	1.29 (1.28–1.31)	1.28 (1.24–1.31)	1.02 (0.99–1.05)
SA- and AV-node dysfunction + pacemaker	1.66 (1.61–1.72)	1.32 (1.24–1.40)	0.83 (0.77–0.90)
Intraventricular conduction blocks	1.47 (1.38–1.57)	1.27 (1.14–1.41)	0.87 (0.76–0.98)
Ventricular tachycardia and fibrillation	1.35 (1.26–1.44)	1.31 (1.17–1.47)	0.97 (0.84–1.11)

	Avg 10-year change in HR	Avg 10-year change in HR	
	Diabetes	Controls	
Change in risk over time as a linear predictor^c^			
Atrial fibrillation and flutter	0.95 (0.93–0.98)	0.91 (0.90–0.93)	
SA- and AV-node dysfunction + pacemaker	1.48 (1.46–1.49)	1.58 (1.57–1.59)	
Intraventricular conduction blocks	1.31 (1.29–1.33)	1.42 (1.41–1.44)	
Ventricular tachycardia and fibrillation	1.16 (1.14–1.18)	1.25 (1.24–1.27)	

The analyses based on Cox regression were adjusted for age, time-updated time periods, sex and interaction terms. Estimates are presented as hazard ratios and 95% confidence intervals.

^a^Excess risk for patients with diabetes and controls in first- and last time periods.

^b^Excess risk for patients with diabetes compared with controls in first- and last time periods. Values are ratios of hazard ratios for patients with type 2 diabetes as compared with during a 10-year period. Values below 1.0 indicates that lesser event-rate reduction.

^c^Excess risk for patients with diabetes and controls, during a 10-year interval, separately.

#### Sinus node disease, atrioventricular node block or pacemaker implantation

The incidence of SND, AV block or pacemaker implantation in people with type 2 diabetes increased from 103 to 118 cases per 100,000 person-years from the first to final time period (Fig. 1B). Corresponding figures for controls were 82 to 72 cases per 100,00 person-years. Patients with type 2 diabetes displayed a 17% (HR 0.83, 95% CI 0.77–0.90) greater relative rate reduction, compared with matched controls (Table 2b). Hazard ratio for a 10-year period showed an 8% risk reduction (HR 0.92, 95% CI 0.87–0.97) (Table 2).

#### Intraventricular conduction block

The incidence of these IVCB increased from 23 to 57 cases per 100,000 person-years in type 2 diabetes and from 10 to 38 cases per 100,000 person-years in controls (Fig. 1C). Patients with type 2 diabetes had a 13% (HR 0.87, 95% CI 0.76–0.98) greater relative rate reduction, compared with controls. During a 10 year period, hazard ratio increased for 14% (HR 1.14, 95% CI 1.07–1.22) (Table 2) among matched controls, without a significant trend for patients with diabetes.

#### Ventricular tachycardia or ventricular fibrillation

The risk of VT/VF in people with type 2 diabetes, compared with controls (Fig. 1D), did not differ during the first and final time periods. The incidence rate was unchanged from 49 to 51 cases per 100,000 person-years in type 2 diabetes and 33 to 32 cases per 100,000 person-years in controls. Hazard ratio for a 10-year period showed that the matched controls had 18% lower risk (HR 0.82, 95% CI 0.77–0.87).

### Association between risk factor control and risk of arrhythmia

**Figure 2:**
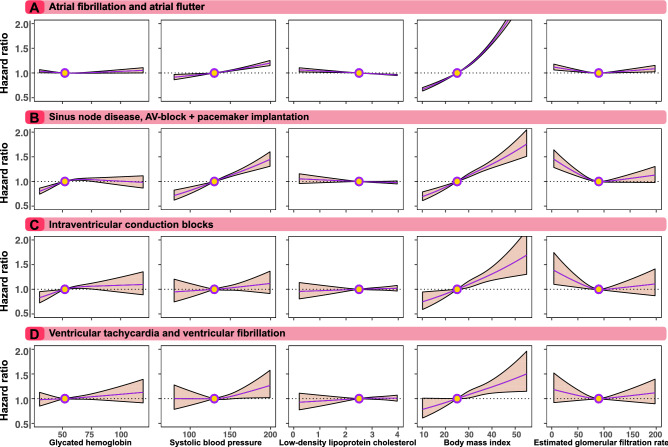
Association between levels of cardiometabolic risk factors for cardiac arrhythmias in patients with type 2 diabetes. We constructed a Cox model for each outcome and applied a prediction function to assess the relationship between selected risk factors and outcomes (Panel A to Panel D). The dark lines indicate the hazard function and the shaded areas 95% confidence intervals. Continuous variables were modeled with restricted cubic splines. The following cut-of levels were used for risk factors: glycated hemoglobin (≥ 7.0% [≥ 53 mmol/mol]), SBP (≥ 130 mmHg), LDL–C (≥ 2.5 mmol/L [97 mg per deciliter]), BMI ≥ 25 kg/m2 and eGFR ≥ 90 mL/min/1.73m2.

Figure 2 shows the associations between glycated haemoglobin, systolic blood pressure, LDL-C, BMI and eGFR and risk of arrhythmia in patients with type 2 diabetes, with diabetes guideline target level depicted with a circle. Figure 2A shows that the association between glycated haemoglobin and the risk of AF/AFl in an adjusted Cox model. The risk of AF/AFl increased linearly with increasing systolic blood pressure and with increasing BMI. LDL-C showed an inverse linear association. Increasing eGFR was associated with a lower risk of AF/AFl.

Overall, similar associations were noted for the risk of SND, AV-block and pacemaker implantation (Fig. 2B); IVCB (Fig. 2C); and VT/VF (Fig. 2D). For eGFR, the risk of VT/VF had a greater magnitude of estimated association at levels below 90 mL/min/1.72 m^2^ (Fig. 2D), although the association was non-significant. Additionally, for SND, AV-block, pacemaker implant and IVCB, glycated haemoglobin levels below target guideline levels were associated with significantly lower risk.

**Figure 3:**
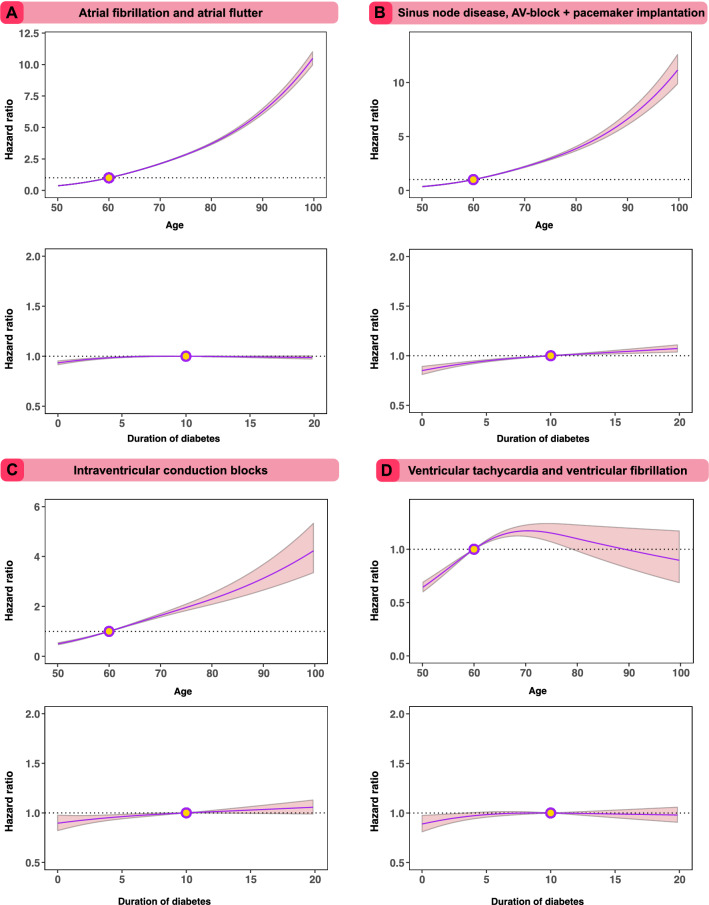
Hazard function for age and duration of diabetes for arrhythmias in patients with type 2 diabetes. Hazard risk for age at baseline and duration of diabetes using restricted cubic splines in an extensively adjusted Cox model. The dark lines indicate the hazard function and the shaded areas 95% confidence intervals.

An additional Cox model, using a similar modelling approach as for the analyses of optimal risk factor levels, shows a substantial risk associated with increasing age at registry baseline and miniscule risk associated with increasing duration of diabetes in patients with type 2 diabetes (Fig. 3).

#### Multifactorial risk factors at target

**Figure 4:**
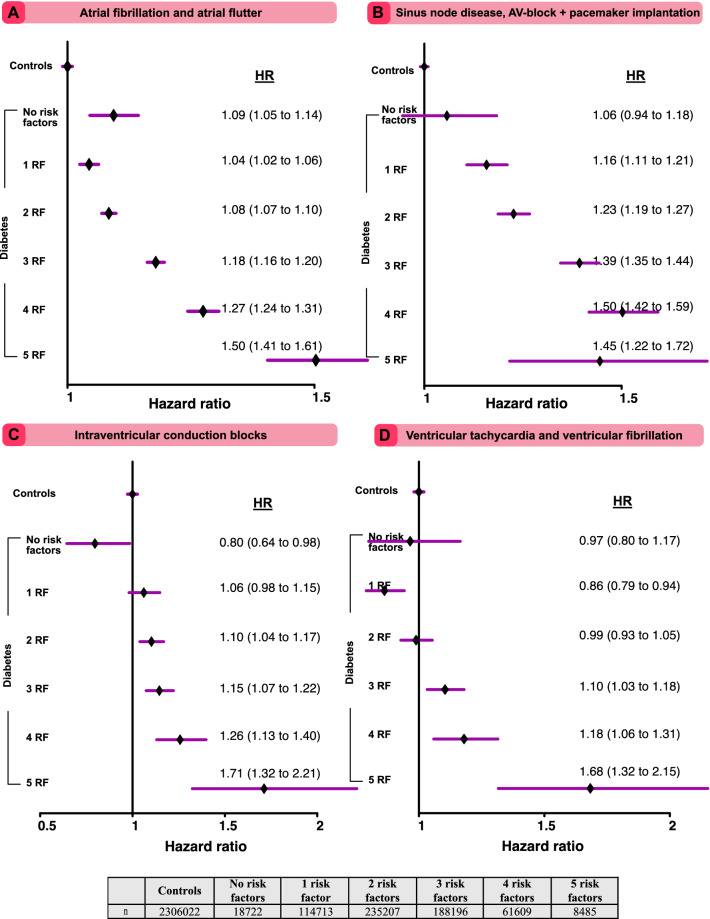
Adjusted hazard ratios for cardiac arrhythmias, according to number of risk factor variables outside target range among patients with type 2 diabetes, as compared to matched controls. Hazard ratios shows the excess risk of each outcome among patients with diabetes, compared to matched controls from the general population, according to number of risk factors (scale, none to five) that were outside therapeutic ranges. Tables displays number of study participants in each risk factor category. The dark lines indicate the hazard function and the shaded areas 95% confidence intervals.

Figure 4A–D shows adjusted hazard ratios for all arrhythmia/conduction outcomes, comparing participants with diabetes in 5 groups of numbers of risk factors at target with matched controls. Throughout, the risk of all arrhythmia outcomes was incrementally higher for each risk factor beyond target. People with type 2 diabetes with 5 risk factors outside of target at baseline displayed a HR of 1.50 (95% CI 1.41–1.61), compared with controls, while having all risk factors within target range displayed a HR of 1.09 (95% CI 1.05–1.14; Fig. 4A). Figure 4B shows that having 5 risk factors outside of target and type 2 diabetes conveyed a hazard ratio of 1.45 (95% CI 1.22–1.72) for sinus node disease, AV-block or pacemaker implant, compared with controls. Figure 4C shows that type 2 diabetes with no risk factors was associated with a HR of 0.80 (95% CI 0.64–0.98), compared with controls. For VT/VF, type 2 diabetes with no risk factors outside target was associated with a HR of 0.97 (95% CI 0.80–1.17), compared with controls. Patients with 1 risk factor outside target had significantly lower risk HR 0.86 (95% CI 0.79–0.94), whereas patients with 5 risk factors outside of target had 68% greater risk (HR 1.68, 95% CI 1.32–2.15).

#### Excess risk of arrhythmia outcomes in diabetes vs controls

**Figure 5:**
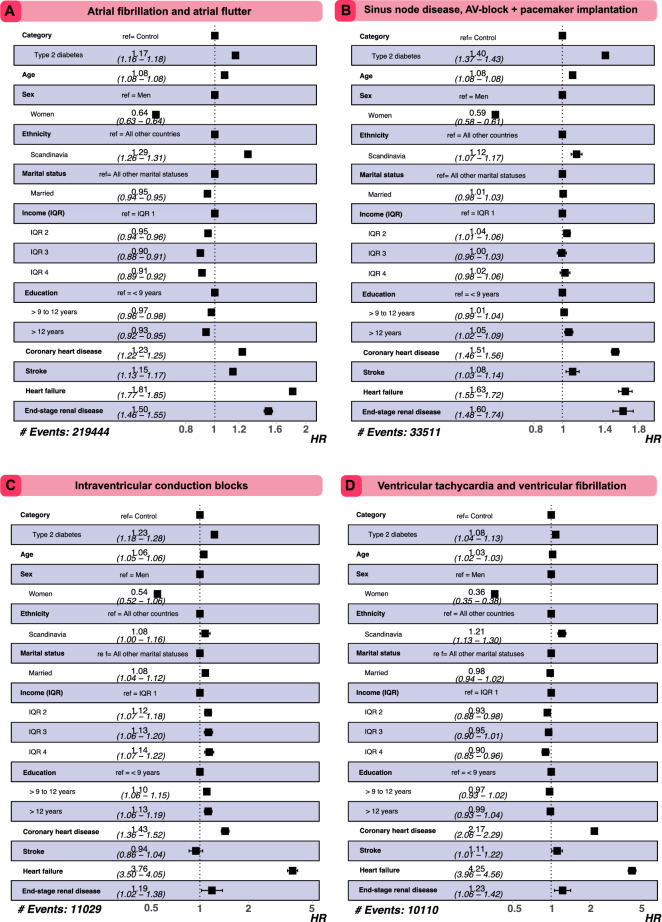
Excess risk for cardiac arrhythmias among patients with type 2 diabetes, as compared to matched controls. Excess risk for arrhythmias was assessed with Cox regression models for patients with diabetes and their matched controls. Inclusion of baseline comorbidities such as coronary heart disease, heart failure and stroke had no significant impact on statin-, hypertensive- and antithrombotic mediation.

Figure 5 shows that overall hazard ratios for people with type 2 diabetes, compared with controls, were 1.23 (95% CI 1.22–1.35) for AF/AFI, 1.45 (95% CI 1.41–1.48) for sinus node disease, AV-block or pacemaker implant, 1.26 (95% CI 1.21–1.31) for IVCB, and 1.20 (95% CI 1.14–1.25) for VT/VF. Women and study participants with Scandinavian ethnicity displayed a lower risk for all outcomes of interest. All cardio-renal baseline comorbidities were associated with increased risk for outcomes, heart failure displayed the highest risk association.

## Discussion

The present analyses of the Swedish National Diabetes Registry data from 2001 through 2019 showed that patients with type 2 diabetes and five selected risk-factor variables within target range had, at most, marginally higher risk for a variety of cardiac arrhythmia complications, including AF/AFl; SA- and AV-node dysfunction or pacemaker implant; IVCB; and VT/VF compared with the general population. The results raise the possibility that having all risk-factor variables within target ranges could theoretically attenuate or even eliminate excess risk for cardiac arrhythmias associated with T2D. We identified a monotonic relationship for increasing number of variables not within target ranges. The following modifiable risk factors were considered to be the independent predictors for cardiac arrhythmias: body mass index, systolic blood pressure and estimated glomerular filtration rate. Using real-world data, we found that levels of glycated hemoglobin and LDL-cholesterol were independently associated with lower risk for cardiac arrhythmias.

Randomized trials investigating the effect of multifactorial cardiometabolic risk-factor intervention on arrhythmia complications in patients with type 2 diabetes are scarce, and contemporary studies have primarily been designed to analyze the independent risk association or cumulative incidence of arrhythmias among patients with various number of risk factors at baseline^6–9^. Additionally, observational studies and randomized trials have shown inconsistent evidence of effects of glycated hemoglobin levels below contemporary guideline levels (< 53 mmol/mol or 7.0%) with regards to atrial fibrillation and flutter^10^. In a previous report from the Swedish National Diabetes Registry, patients with type 2 diabetes and poor glycemic control or kidney function were at higher risk for AF/AFl^11^. However, that study included patients from 2001 to 2013, the analyses were based on individuals with complete data for risk factors and used time-updated glycated haemoglobin levels in the modelling. In the present study, we used baseline values, i.e., risk factor values observed at entry into the Swedish National Diabetes Registry, restricted cubic splines and extensive adjustment of covariates. Ours findings may be limited by the impact of reverse causality, meaning that AF/AFl (i.e. the outcomes) leads to intensification of medical therapy, including glucose-lowering treatments.

The three mechanisms of tachyarrhythmias (automaticity, triggered activity, and re-entry) all depend on the excitability of the conduction cells and the contractile cells in the atria and ventricles. Hyperglycemia causes cellular and extracellular changes, e.g., fibrotic remodeling and inflammation^12^, that may impair cellular function and thus excitability and conduction. It could be perceived that glycemic load (i.e., duration of exposure to hyperglycemia/dysglycemia and glucose fluctuations), would be a more suitable predictor to distinguish the association between glucose and atrial tachyarrhythmias. Our Cox models revealed that duration for diabetes, at baseline, was not a strong risk factor for arrhythmias.

The results from the present study suggest that contemporary risk factor management may have contributed to mitigating risk for SA- and AV-node dysfunction or pacemaker implant and IVCB amongst patients with type 2 diabetes, whereas risk for atrial- and ventricular tachyarrhythmias did not change significantly over time. The trends for reported cardiac arrhythmias show the opposite of trends reported from a related dataset for coronary artery disease, acute myocardial infarction, and heart failure, all of which demonstrated a substantial reduction in incidence and risk, and have been causally linked to the arrhythmias studied here^1,4^.

It is also noteworthy that the overall excess risk of arrhythmia for participants with type 2 diabetes was relatively low, with an excess risk ranging from 8 to 40% increased probability of the outcomes. This could be compared with the excess risk of coronary artery disease, myocardial infarction, stroke, and heart failure that typically range between two- to fourfold in people with type 2 diabetes.

Analyses of risk factors within recommended targets revealed that excess risk for atrial fibrillation and flutter among patients with type 2 diabetes was associated with substantially lower risk by means of optimal risk factor control. However, first, the present analyses evaluated risk factor status independent of treatment; and second, the present analyses evaluate observational associations between baseline level and subsequent outcomes, as opposed to treatment and analyses of time-updated levels. By these limitations, extrapolating to any implication about treatment to affect outcomes has noted limitations. Patients with diabetes and all risk factors within target did not display an excess risk for other arrhythmias, and risk for IVCB was 20% lower compared with matched controls. There is a graded association in arrhythmia risk for each risk factor outside of recommended target, most notably for AF/AFl.

Increasing body mass index was associated with increasing risk for most arrhythmias. Given the impact of increased adiposity on cardiac structure and function, elevated epicardial fat, blood pressure and systemic inflammation, all of which could contribute to arrhythmic risk, obesity remains a promising target for intervention of such risk, independent of T2D status. Further investigation in epicardial adipocyte mass and inflammation that leads to cardiac myopathy is warranted. Previous studies show that interventions such as marked weight loss^13–16^, statin therapy^17–19^ and renin–angiotensin–aldosterone system inhibition^20,21^, have the potential to reduce the risk of AF/AFl as well as the progression of paroxysmal to permanent AF/AFl. Antihyperglycemic medications such as metformin and PPAR-γ inhibitors reduce epicardial adipose tissue and systemic inflammation, whereas insulin promotes adipogenesis and cardiac fibrosis. A similar fibrotic process is perceived to play an important role in apoptosis and fibrosis of sinoatrial- and atrioventricular node disease through independent activation of calmodulin and cellular Ca^2+^ by oxidation, although fibrosis is common but not a universal finding in sinoatrial- and atrioventricular node remodeling and disease. Our analyses show that systolic blood pressure and eGFR are more associated with sinoatrial- and atrioventricular node, and subsequent pacemaker implantation, compared with atrial fibrillation and flutter.

## Limitations

We only assessed diagnoses recorded during inpatient- and outpatient visits, meaning that disease onset may have occurred before the captured event of interest. Nevertheless, previous validations of the Hospital Discharge Registry have proven to be very reliable^22^. We did not have cardiometabolic data on controls, making it impossible to adjust for these risk factors in the regression models. Using baseline values for risk factors may be considered as a limitation, however, index values are preferred due to their advantage from a clinical point of view. Results are model dependent and could change slightly with different approaches to data analyses. Additionally, no distinctions were made between patients with all or some cardiometabolic risk factors within the target range and those treated to guideline target levels. In contrast to AF/AFl, the other composite outcomes are less validated and likely suffers from a lower level of ascertainment in the Swedish patient registries. Additionally, we can’t exclude the possibility of referral bias for the T2D patient group, compared to matched controls, resulting increased detection of cardiac arrhythmias.

## Conclusion

Diabetes mellitus was associated with a higher risk of cardiac arrhythmias and conduction disturbances, although patients with type 2 diabetes with cardiovascular risk factors within target had little or no excess risk of cardiac arrhythmias. Incremental risks for atrial fibrillation and flutter, as well as ventricular tachycardia and fibrillation, associated with T2D have not changed significantly compared with matched controls, whereas intraventricular conduction blocks and sinus node disease, AV-block or pacemaker implant have decreased significantly compared with controls.

## Supplementary Information

Supplementary Information.

## Data Availability

The datasets generated during and/or analysed during the current study are not publicly available since they are provided by three different Swedish government agencies, such as The Swedish social welfare board (email: socialstyrelsen@socialstyrelsen.se), The Swedish Association of Local Authorities and Regions (email: info@skr.se and ndrinfo@registercentrum.se) and Statistics Sweden (scb@scb.se). All researchers can apply for these datasets by contacting these government agencies and by fulfilling legal and regulatory requirements and providing their acceptance letter from the Swedish Ethical Review Authority of Ministry of Sweden. Thus, these datasets are not available from the corresponding author on reasonable request.
