# Supplementary material for: Cardiac arrhythmias and conduction abnormalities in patients with type 2 diabetes
Source: Sci Rep. 2023 Jan 21;13:1192. doi: 10.1038/s41598-023-27941-5 (PMC9867726; doi:10.1038/s41598-023-27941-5)
Supplement: Supplementary file 1 — Supplementary Information. [file 41598_2023_27941_MOESM1_ESM.pdf]

Cardiac Arrhythmias and Conduction Abnormalities in Type 2 Diabetes

Supplemental material

Supplementary discussion of the statistical methods

This is a brief discussion regarding the statistical models used in this study. For this study, we calculated crude- and standardized incidence rates for outcomes, among patients with diabetes and controls. In addition, we applied Cox proportional hazards model excess risk between the first- and last time periods, in first- and last time periods. Additionally, Cox regression was used to assess the relative risk associated with the number of risk factors at target for patients with diabetes, compared to matched controls, and to assess optimal levels for five selected risk factors, by applying a prediction function to our regression models.

Incidence rates and change in risk over time

For the standardized incidence rates, we used Swedish population life tables, from the initial time period as the reference population for the type 2 diabetes case control cohort. We constructed the following age-categories, **1. < 45, 2. 45–54, 3. 55–64, 4. 65–74 and 5. > 75** years of age, for the reference population and our study cohort. The reference population was also stratified on gender.

<u>The following Cox regression model was used separately for both the first and last time periods:</u>

Table 2: [a] First- and last time period (example)

$$\lambda(survtime_{atrial\,fibrillation\,\&\,flutter},\ event_{atrial\,fibrillation\,\&\,flutter}) = \exp(\beta_1 category_{j1}) + \beta_2 age_{j2} + \beta_3 sex_{j3} + \beta_3 ethnicity_{j3} + \beta_4 marital\ status_{j4} + \beta_5 income\_quartiles_{j5} + \beta_6 education_{j6}$$

Table 2: [b] Diabetes vs controls (first- vs last time period)

$$\lambda(survtime_{atrial\,fibrillation\,\&\,flutter},\ event_{atrial\,fibrillation\,\&\,flutter}) = \beta_1 acategory_{j1} + \beta_2 age_{j2} + \beta_3 sex_{j3} + \beta_4 timeperiod_{j4} + exp(\beta_5 category * timeperiod)_{j5} + \beta_6 (category * age)_{j6} + \beta_7 (category * sex)_{j7}$$

Table 2: [c] 10-year hazard ratio

$$\lambda(survtime_{atrial\,fibrillation\,\&\,flutter},\ event_{atrial\,fibrillation\,\&\,flutter}) = exp(\beta_1 timeperiod_{j1}) + \beta_2 age_{j2} + \beta_3 sex_{j3} + \beta_3 ethnicity_{j3} + \beta_4 marital\ status_{j4} + \beta_5 income\_quartiles_{j5} + \beta_6 education_{j6}$$

The variable *category* denotes either patients with diabetes or matched control. Time-period indicated either first- or last time period, however, in the final model, time period was modeled as a linear term. The coefficient for this variables shows the average relative risk reduction for a 2-year interval, therefore exponentiating this coefficient with 5 results in the average 10-year change in hazard ratio.

Multifactorial risk factor control

We constructed a Cox regression model that includes number of risk factors at target ranging from 0 to 5 (referred to as ***β_1(category)***), modeled as categorical variables. For this variable,

we included traditional and modifiable cardiometabolic risk factors such as, glycated hemoglobin, systolic- and diastolic blood pressure, low-density lipoprotein cholesterol, smoking and renal function (albuminuria).

In order to achieve even greater statistical power in these analyses, we decided to categorize the ***β_1(category)*** variable even further, ranging between 2–3 categories in the final models. Matched controls had a duration of diabetes set to zero years and were also considered the reference *group* for the ***β_1(category)*** variable, whereas patients with diabetes had their duration of diabetes subtracted with the grand mean of the respective diabetes cohorts.

All Cox models are adjusted for age, sex, socioeconomic variables, baseline comorbidities and treatment with either antihypertensives, statins, antithrombotic- or anticoagulant medication. An example of the Cox model for the analysis of number of risk factor at target is depicted below.

<u>Figure 4: Example of Cox model for atrial fibrillation and atrial flutter</u>

$$\begin{aligned}
&\lambda(survtime_{atrial\,fibrillation\,\&\,flutter},\ event_{atrial\,fibrillation\,\&\,flutter})\\
&= exp(\beta_1 category_{j1}) + \beta_2 diabetesduration_{j2} + strata(\beta_3 sex_{j3})\\
&+ \beta_4 income_quartiles_{j4} + \beta_5 eduation_{j5} + \beta_6 maritalstatus_{j6} + \beta_7 ethnicity_{j7}\\
&+ \beta_8 pre_chd_{j8} + \beta_9 pre_heartfailure_{j9} + \beta_{10} pre_atrialfibrillation_{j10}\\
&+ \beta_{11} pre_stroke_{j11} + \beta_{12} pre_copd_{j12} + \beta_{13} pre_dementia_{j13}\\
&+ \beta_{14} pre_hypertension_{j14} + \beta_{15} pre_renal_{j15} + \beta_{16} pre_cancer_{j16}\\
&+ \beta_{17} antihypertensives_{j17} + \beta_{18} statins_{j18} + \beta_{19} antithrombotic_{j19}\\
&+ \beta_{20} antitcoagulants_{j20} + \beta_{21} age_{j21}
\end{aligned}$$

Optimal levels for risk factors

We used the general Cox model with restricted cubic splines to delineate the value that was associated with the lowest risk for each outcome. The guideline target level was set as reference level for each risk factor. For glycated hemoglobin we used 53 mmol/mol (7.0%), for systolic blood pressure 130 mmHg and for low-density lipoprotein cholesterol 2.5 mmol/L.

For continuous variables, we used restricted cubic splines with three evenly spaced knots and did experiment with additional splines and knots for all continuous predictors, however between 2 to 3 knots seemed optimal, 4 and more did not contribute especially much to the model.

This model serves as an example of how our Cox models were constructed and glycated hemoglobin was predicted:

<u>Figure 2: Example of Cox model for atrial fibrillation and atrial flutter (Type 2 Diabetes)</u>

$$\lambda(survtime_{atrial\,fibrillation\,\&\,flutter},\ event_{atrial\,fibrillation\,\&\,flutter}) \\ = \times\ exp(rcs(\beta_1 HbA1c_{j1}, 3) + strata(\beta_2 sex_{j2}) + rcs(\beta_3 BMI_{j3}, 3) \\ + rcs(\beta_4 LDL - C_{j4}, 3) + rcs(\beta_5 HDL - C_{j5}, 3) + rcs(\beta_6 eGFR_{j6}, \\ 3) + rcs(\beta_7 Age_{j7}, 3) + rcs(\beta_8 systolicBP_{j8}, 3) + rcs(\beta_9 diastolicDP_{j9}, 3) \\ + \beta_{10} physical\_acitivity_{j10} + \beta_{11} pre\_chd_{j11} + \beta_{12} pre\_heartfailure_{j12} \\ + \beta_{13} pre\_atrialfibrillation_{j13} + \beta_{14} income\_quartiles_{j14} + \beta_{15} education_{j15} \\ + \beta_{16} ethnicity_{j16} + \beta_{17} statins_{j17} + \beta_{18} antihypertensives_{j18} + \beta_{19} smoking_{j19} \\ + \beta_{20} antithrombotics_{j20} + \beta_{21} anticoagulants_{j21} + \beta_{22} pre\_stroke_{j22} \\ + \beta_{23} pre\_hypertension_{j23} + \beta_{24} pre\_pad_{j24} + \beta_{25} pre\_copd_{j25} \\ + \beta_{26} pre\_dementia_{j26} + \beta_{27} pre\_renal_{j27} + \beta_{28} pre\_cancer_{j28} \\ + rcs(\beta_{29} diabetesduration_{j29}, 3)$$

In Figure 3, we have used a similar regression model to estimate risk associated with age at baseline and duration of diabetes.

Excess risk for cardiac arrhythmias

We constructed Cox regression models to estimate excess risk for arrhythmias, in the entire diabetes cohorts, compared to their matched controls. As seen in Figure 5 in main manuscript, we included the following variables:

<u>Figure 5: Example of atrial fibrillation and atrial flutter</u>

$$\lambda(survtime_{atrial\,fibrillation\,\&\,flutter},\ event_{atrial\,fibrillation\,\&\,flutter}) \\ = \exp(\beta_1 category_{j1}) + \beta_2 age_{j2} + \beta_3 sex_{j3} + \beta_3 ethnicity_{j3} + \beta_4 marital\ status_{j4} \\ + \beta_5 income\_quartiles_{j5} + \beta_6 education_{j6}$$

The variable *category* denotes either patients with diabetes or matched control

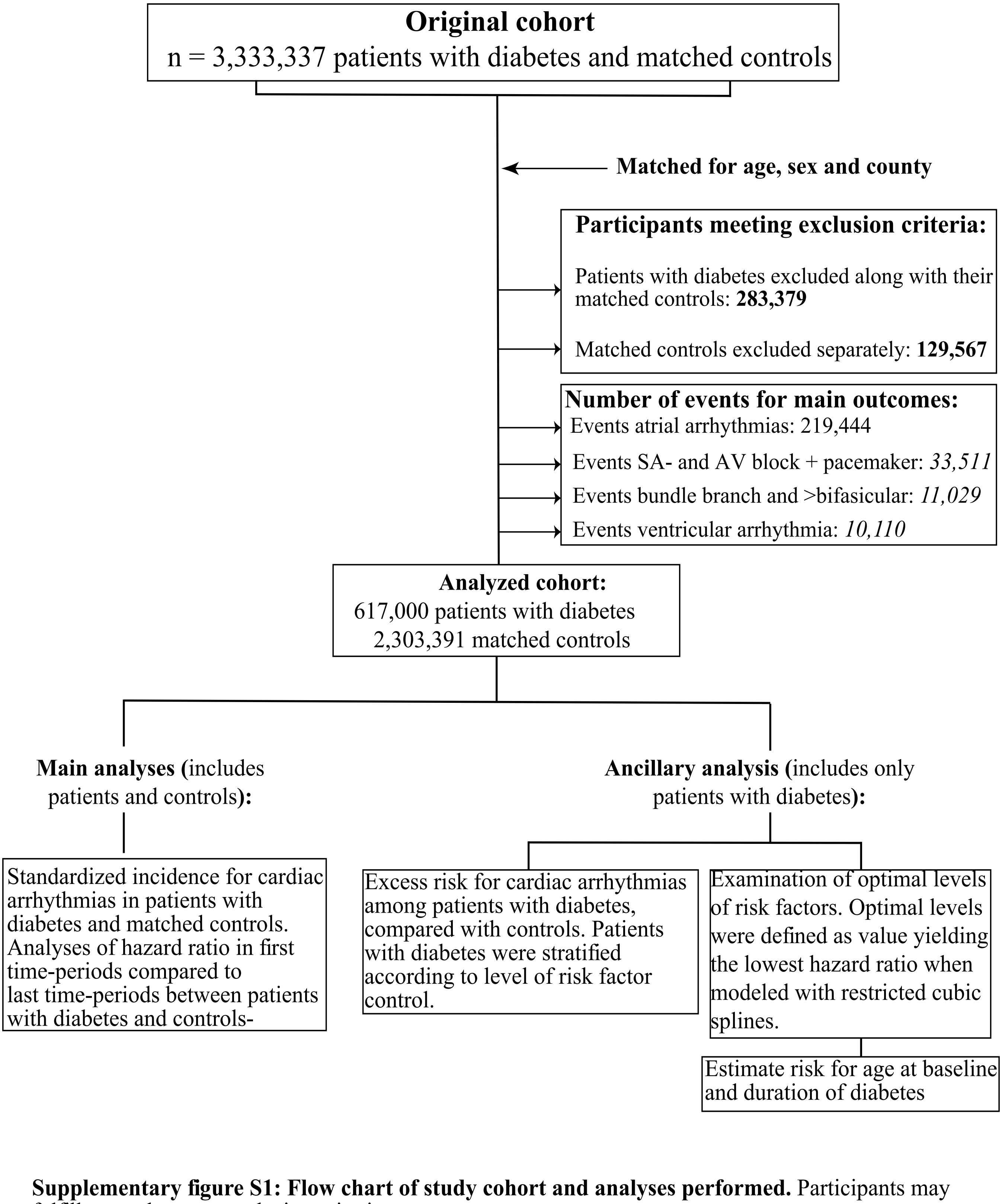

Supplementary figure S1: Flow chart of study cohort and analyses performed. Participants may fulfill more than one exclusion criteria

Table S1. ICD-9 and ICD-10 Codes Used to Define Baseline Conditions and Outcomes		
**Outcomes**	**ICD-9**	**ICD-10**
**Cardiac arrhythmias**		
Atrial fibrillation and atrial flutter	I427D, I4273	I480, I481, I482, I483, I484, I489
Sinus node disease, atrioventricular block and cardiac pacemaker implantation	I426A, I4260, V45A, V450	I441, I442, I495, Z950
Intraventricular conduction blocks	I426C, I426D, I4263, I426E, I4264, I426F, I426G, I426	I444, I445, I457, I450, I451, I452, I453
Ventricular tachycardia and ventricular fibrillation	I427B, I4271, I427E, I4274,	I490, I472
**Comorbidities**		
Coronary heart disease	410-414	I20-I25
Stroke	431-434, 436	I61-I64
Heart failure & cardiomyopathies	425, 421, 428	I50, I42
Hypertension	401, 402, 403, 404, 405	I109, I11, I120, I129, I132, I139, I130, I131, I159, I150, I152, I158
Peripheral arterial disease	440C, 443X	I702, I739
Dementia	294B	F00, F01, F02, F039
End-stage renal disease	5818. 584, 585, 583, 590, 586, 593X, 791X, V42A, 250D, 403, V45B, V56	N179, N178, N170, N172, N189, N181, N182, N183, N184, N185, N199, Z940, DR016, N083, E112C, E112C, E112W, E112X, E102, E102X, E132, E242, E122, E112A, E112B, I120, Z992, DR013, DR023, DR056, Z941, Z492
Cancer		C00–C97
COPD	496, 491	J44
Alcoholism	291, 292, 303, 305A	F10
* Includes ICD-codes as main diagnosis and up to 6 contributory causes for outcomes and comorbidities		

Table S2. Crude- and standardized incidence rates for atrial fibrillation/flutter and SA- AV node dysfunction and pacemaker implantation, in patients with type 2 diabetes and matched controls for all time-periods.

Crude rate	Adjusted rate	Lower confidence interval	Upper confidence interval	Events	Person-years	Time-period	n	Category
**Atrial fibrillation and flutter**								
1535,7	780,6	682,7	922,2	468	30474,4	P1	88084	Diabetes
1482	701,5	663,6	751,7	1634	110258,7	P2	266642	Diabetes
1437,7	693,4	662,9	730,2	3103	215825,4	P3	489196	Diabetes
1496,4	729	701,3	760,8	5162	344954	P4	763224	Diabetes
1513,3	731,2	709	756,2	7417	490134,8	P5	1059825	Diabetes
1549,2	743	723	765,1	9421	608118,6	P6	1276934	Diabetes
1532,4	721,4	702,7	741,9	10354	675650,4	P7	1408809	Diabetes
1503,3	703,5	685,4	723,3	11075	736722,5	P8	1533857	Diabetes
1497,7	676,2	663,7	689,8	18350	1225246	P9	2536505	Diabetes
1279,8	661,9	621,6	710	1258	98296	P1	285246	Control
1127,2	594,6	574,6	616,9	3915	347329,5	P2	843936	Control
1038,9	564,4	549,9	580,1	7055	679081	P3	1543475	Control
987,5	548,2	536,9	560,3	10883	1102054	P4	2444784	Control
948,1	544,1	534,3	554,5	15355	1619639	P5	3508060	Control
928,4	526,7	518,5	535,4	19493	2099669	P6	4409042	Control
933,5	522,8	514,8	531,1	22820	2444499	P7	5091715	Control
916,8	495,3	488,3	502,6	25661	2799107	P8	5821763	Control
926,2	477,8	472,8	483	46019	4968742	P9	10265575	Control
**Sinus node disease, AV-block and pacemaker**								
224,7	103	79,4	189,3	69	30708	P1	88315	Diabetes
174,1	77,5	66,6	105,9	195	111986,5	P2	269485	Diabetes
204,4	89,8	81,3	106,9	451	220647,2	P3	497854	Diabetes
211,9	91,9	85,2	104	752	354872,3	P4	781494	Diabetes
225,1	101,2	93,6	112,3	1142	507236,4	P5	1091967	Diabetes
249,3	114,1	106,4	124,4	1580	633687,6	P6	1325871	Diabetes
264,1	118,8	111,5	128,3	1874	709597,4	P7	1474639	Diabetes
265,8	113,1	106,3	121,9	2069	778508,7	P8	1615553	Diabetes
279,6	118,7	112,9	125,6	3641	1302212	P9	2687456	Diabetes
135,5	82,7	63,5	112,1	134	98893,8	P1	285844	Control
121,9	66,8	59	77,3	428	351205	P2	850373	Control
120,1	66,6	61,1	73,5	828	689531,4	P3	1562257	Control
118,2	64,7	60,9	69,5	1326	1122223	P4	2482163	Control
113,2	63,1	59,9	67	1872	1653942	P5	3572375	Control
124,1	69,6	66,6	73,1	2670	2151872	P6	4508557	Control
128,2	70,7	67,8	74	3227	2517540	P7	5232265	Control
131,2	70,3	67,5	73,4	3797	2894941	P8	6007374	Control
144,4	72,1	70,1	74,2	7456	5161962	P9	10641236	Control
**Intraventricular conduction blocks**								
48,8	22,5	12,2	110,3	15	30745,8	P1	88359	Diabetes

50,8	23	17,2	50	57	112218,8	P2	269873	Diabetes
56	26,4	21,6	41,6	124	221271,1	P3	498956	Diabetes
58,7	27,6	23,6	38	209	356204,2	P4	783963	Diabetes
73	40,6	33,3	51,7	372	509512	P5	1096259	Diabetes
78	38,6	33,3	46,7	497	637260,2	P6	1332647	Diabetes
84,5	44,6	39,2	52,6	604	714647,1	P7	1484327	Diabetes
77,6	36,8	32,8	43,2	609	785110,7	P8	1628368	Diabetes
82,1	37,2	34,3	41,5	1079	1314734	P9	2712006	Diabetes
18,2	9,5	5,5	27,8	18	98954,3	P1	285890	Control
26,7	14,7	11,7	21,1	94	351671,8	P2	851130	Control
33,4	19,1	16,3	23,6	231	690708,9	P3	1564371	Control
34,8	20,2	17,8	23,7	391	1124587	P4	2486536	Control
40,9	23,4	21,5	25,9	678	1657844	P5	3579781	Control
45	27,9	25,5	30,8	970	2157816	P6	4519926	Control
48,3	27,1	25,2	29,3	1220	2525995	P7	5248546	Control
46,8	26	24,3	28,1	1361	2906137	P8	6029041	Control
48,2	25,8	24,4	27,3	2500	5185486	P9	10686727	Control
Ventricular tachycardia and fibrillation								
81,3	47,5	27,9	136,1	25	30740,3	P1	88355	Diabetes
61,5	32,2	24	60,1	69	112208,5	P2	269854	Diabetes
58,7	35,3	26,2	53,9	130	221277,6	P3	498960	Diabetes
62,3	32,6	27,8	43,7	222	356216,8	P4	783966	Diabetes
61,2	31,9	26,9	40,8	312	509652,9	P5	1096491	Diabetes
71,4	39,5	33,5	48,4	455	637517,9	P6	1333134	Diabetes
69,5	41,1	34,3	50,7	497	715124	P7	1485247	Diabetes
71,5	36,8	32	44	562	785744,5	P8	1629630	Diabetes
68,3	42,4	36,4	50	899	1315909	P9	2714325	Diabetes
59,6	31,4	23,8	50,7	59	98936,6	P1	285869	Control
58,3	40,4	32,8	51,2	205	351579,7	P2	850988	Control
48,1	29,5	25,5	35,2	332	690563,3	P3	1564084	Control
41,4	24,9	22,3	28,5	466	1124461	P4	2486269	Control
42	27,4	24,5	31	696	1657794	P5	3579621	Control
41,3	25,1	23	27,7	892	2158036	P6	4520292	Control
41,2	25	23	27,5	1042	2526660	P7	5249734	Control
40	24,7	22,5	27,2	1162	2907226	P8	6031100	Control
40,2	23,3	21,9	25	2085	5187907	P9	10691372	Control

Table S3. Crude- and standardized incidence rates for atrial fibrillation/flutter according to age-categories, in patients with type 2 diabetes and matched controls for all time-periods.									
Crude rate	Adjusted rate	Lower confidence interval	Upper confidence interval	Events	Person-years	Time-period	n	Category	Age-groups
157,3	108,1	42,2	426,4	3	1907,3	P1	4868	Diabetes	(18-44)
15,3	11,3	1,6	82,5	1	6541,7	P2	14674	Diabetes	(18-44)
77,1	94,2	57,7	150,1	10	12965,9	P3	28030	Diabetes	(18-44)
97,5	103,9	75,7	141,2	21	21541,3	P4	46099	Diabetes	(18-44)
117,1	135,3	108,9	167,1	39	33306,4	P5	70205	Diabetes	(18-44)
118	81,2	65,6	682,5	54	45749,5	P6	94304	Diabetes	(18-44)
119,8	163,9	81,3	331,4	67	55911,7	P7	114500	Diabetes	(18-44)
137,2	160,1	116,6	229,7	91	66318,9	P8	135616	Diabetes	(18-44)
145,8	219,2	185	260,5	175	120051	P9	243637	Diabetes	(18-44)
56	68,5	26,4	167,4	5	8936,4	P1	22775	Control	(18-44)
51,6	84,7	57,3	123	16	30983,2	P2	69512	Control	(18-44)
40,5	49,6	36,7	66,4	25	61742,3	P3	133517	Control	(18-44)
57,1	72,8	60,9	86,7	59	103322,3	P4	221279	Control	(18-44)
65,7	76,5	67,2	86,9	106	161325,6	P5	340215	Control	(18-44)
57,5	204,8	107,9	386,6	129	224341,6	P6	462741	Control	(18-44)
68,8	113,1	84,8	154,9	191	277460,8	P7	568512	Control	(18-44)
78,7	110,4	93,1	132,5	262	332887,7	P8	680943	Control	(18-44)
89	119,3	108,7	131,2	545	612514,9	P9	1243365	Control	(18-44)
511,4	495,9	341,3	747,8	62	12123,1	P1	34048	Diabetes	(45-64)
562,9	599,1	515,8	697,2	257	45657,7	P2	108387	Diabetes	(45-64)
612,8	586,2	537,8	639,6	571	93181,3	P3	208031	Diabetes	(45-64)
635,3	593,6	557,3	632,4	977	153781,5	P4	335094	Diabetes	(45-64)
712,6	646,6	616,6	678,2	1602	224794,8	P5	479420	Diabetes	(45-64)
757,8	844,1	673,9	1074,4	2170	286347,4	P6	594777	Diabetes	(45-64)
805,4	842,7	766,7	927,9	2650	329036,8	P7	679138	Diabetes	(45-64)
859,2	794,8	749,5	843,2	3174	369410,6	P8	761446	Diabetes	(45-64)
912,6	810,8	784	838,5	5847	640662,5	P9	1320860	Diabetes	(45-64)
353,9	485,9	390,5	607,5	148	41822,7	P1	117590	Control	(45-64)
340,3	376,3	341,4	415,2	536	157501,7	P2	375803	Control	(45-64)
339,2	366,4	345,1	389	1111	327498,8	P3	735047	Control	(45-64)
363,7	361,8	346,9	377,4	2025	556822,5	P4	1219693	Control	(45-64)
379,1	377,3	365,6	389,5	3203	844865,5	P5	1810142	Control	(45-64)
412,7	519,8	436,3	620,9	4613	1117686	P6	2327201	Control	(45-64)
458	496,5	464,2	531,2	6080	1327523	P7	2743826	Control	(45-64)
487,1	488,1	468,7	508,5	7512	1542327	P8	3182778	Control	(45-64)
555,7	528,7	517,7	539,8	15523	2793396	P9	5759055	Control	(45-64)
2291,5	2515,6	2336,1	2707,3	343	14968,2	P1	44494	Diabetes	(65-84)
2251,7	2338,9	2248,5	2432,6	1205	53515,6	P2	131631	Diabetes	(65-84)
2105,8	2167	2104,1	2231,6	2152	102191,6	P3	234664	Diabetes	(65-84)
2307	2332,8	2280,7	2386	3666	158909,6	P4	355907	Diabetes	(65-84)
2315,7	2275,3	2231,7	2319,8	5040	217645	P5	475622	Diabetes	(65-84)

2441,3	2366,5	2325,9	2407,9	6357	260393,1	P6	552345	Diabetes	(65-84)
2508,4	2352,2	2313	2392,2	6949	277026,1	P7	584227	Diabetes	(65-84)
2501,3	2284	2246	2322,7	7229	289006,1	P8	609543	Diabetes	(65-84)
2565,7	2292,2	2261	2323,8	11526	449239,8	P9	937712	Diabetes	(65-84)
2133,9	2308,3	2202,6	2418,5	920	43113,9	P1	130503	Control	(65-84)
1929,7	2056,3	2002	2112	2820	146137,8	P2	364748	Control	(65-84)
1880,9	1999,5	1959,8	2039,9	5084	270297,5	P3	626232	Control	(65-84)
1849,8	1955,4	1923,6	1987,6	7690	415719,9	P4	939116	Control	(65-84)
1820,7	1930,7	1903,8	1957,9	10547	579282,4	P5	1274973	Control	(65-84)
1810,1	1903,8	1879,9	1928	13036	720183,8	P6	1533842	Control	(65-84)
1867,2	1875,7	1853,6	1898,2	15052	806125,1	P7	1703707	Control	(65-84)
1849,5	1830,7	1810	1851,6	16532	893864,6	P8	1889754	Control	(65-84)
1852,1	1761,6	1746,2	1777,2	28189	1522022	P9	3171783	Control	(65-84)
4065,6	4165,5	3639,6	4768,4	60	1475,8	P1	4674	Diabetes	(>85)
3763,4	3881,9	3587,3	4201,1	171	4543,7	P2	11950	Diabetes	(>85)
4942,2	5009,5	4749,6	5284	370	7486,6	P3	18471	Diabetes	(>85)
4644,8	4793,9	4579,5	5018,5	498	10721,7	P4	26124	Diabetes	(>85)
5115,2	5354,8	5156,9	5560,3	736	14388,6	P5	34578	Diabetes	(>85)
5374,7	5543,9	5350,5	5744,5	840	15628,7	P6	35508	Diabetes	(>85)
5030,8	5210,7	5009,2	5420,6	688	13675,8	P7	30944	Diabetes	(>85)
4846,9	4929,3	4722,9	5144,9	581	11987	P8	27252	Diabetes	(>85)
5244,3	5403,7	5211,4	5603,3	802	15292,9	P9	34296	Diabetes	(>85)
4182,7	4473,2	4135,6	4839,2	185	4423	P1	14378	Control	(>85)
4273,3	4423	4226	4629,6	543	12706,8	P2	33873	Control	(>85)
4272,8	4525,9	4362	4696,1	835	19542,4	P3	48679	Control	(>85)
4234,6	4453,3	4312,6	4598,9	1109	26189,1	P4	64696	Control	(>85)
4387,5	4762,3	4631,3	4897,2	1499	34165,4	P5	82730	Control	(>85)
4578,5	4880,5	4753,3	5011,3	1715	37457,8	P6	85258	Control	(>85)
4483,3	4858,8	4722	4999,8	1497	33390,3	P7	75670	Control	(>85)
4512,5	4782,5	4640,4	4929,3	1355	30027,7	P8	68288	Control	(>85)
4317,6	4674,8	4553,3	4799,8	1762	40809,4	P9	91372	Control	(>85)

Table S4 Variables Used in the Imputation Algorithm.

Age, sex, age at onset of diabetes, clinicians diagnosis type, epidemiological definition of diabetes, treatment of diabetes, method of insulin treatment, systolic blood pressure, diastolic blood pressure, body weight, body length, glycated hemoglobin (HbA1c), total cholesterol, triglycerides, HDL-cholesterol, LDL-cholesterol, albuminuria, s-creatinine, retinopathy, smoking status, physical activity, county, body mass index, marital status, education, ethnicity, income, family income, eGFR, history of acute myocardial infarction, coronary heart disease, heart failure, hypertension, peripheral arterial disease, chronic obstructive pulmonary disease, dementia, alcoholism, end-stage renal disease, cancer, stroke, treatment with anti-hypertensive medication, statins, antithrombotic and anti-coagulant medication.
